# Altered Protein Expression in Gestational Diabetes Mellitus Placentas Provides Insight into Insulin Resistance and Coagulation/Fibrinolysis Pathways

**DOI:** 10.1371/journal.pone.0044701

**Published:** 2012-09-07

**Authors:** Bin Liu, Yun Xu, Courtney Voss, Fang-hua Qiu, Ming-zhe Zhao, Yong-dong Liu, Jing Nie, Zi-lian Wang

**Affiliations:** 1 Department of Obstetrics and Gynecology, The First Affiliated Hospital of Sun Yat-sen University, Guangzhou, People’s Republic of China; 2 Division of Nephrology, Nanfang Hospital, Southern Medical University, Guangzhou, People’s Republic of China; 3 Department of Endocrinology, The Sixth Affiliated Hospital of Sun Yat-sen University, Guangzhou, People’s Republic of China; 4 Department of Biochemistry, University of Western Ontario, London, Ontario, Canada; 5 Lab of Proteomics, The First Affiliated Hospital of Sun Yat-sen University, Guangzhou, People’s Republic of China; 6 Department of Pathology, The First Affiliated Hospital of Sun Yat-sen University, Guangzhou, People’s Republic of China; VU University Medical Center, The Netherlands

## Abstract

**Objective:**

To investigate the placental proteome differences between pregnant women complicated with gestational diabetes mellitus (GDM) and those with normal glucose tolerance (NGT).

**Methods:**

We used two-dimensional electrophoresis (2DE) to separate and compare placental protein levels from GDM and NGT groups. Differentially expressed proteins between the two groups were identified by MALDI-TOF/TOF mass spectrometry and further confirmed by Western blotting. The mRNA levels of related proteins were measured by realtime RT-PCR. Immunohistochemistry (IHC) was performed to examine the cellular location of the proteins expressed in placenta villi.

**Results:**

Twenty-one protein spots were differentially expressed between GDM and NGT placenta villi in the tested samples, fifteen of which were successfully identified by mass spectrometry. The molecular functions of these differentially expressed proteins include blood coagulation, signal transduction, anti-apoptosis, ATP binding, phospholipid binding, calcium ion binding, platelet activation, and tryptophan-tRNA ligase activity. Both protein and mRNA levels of Annexin A2, Annexin A5 and 14-3-3 protein ζ/δ were up-regulated, while the expression of the Ras-related protein Rap1A was down-regulated in the GDM placenta group.

**Conclusion:**

Placenta villi derived from GDM pregnant women exhibit significant proteome differences compared to those of NGT mothers. The identified differentially expressed proteins are mainly associated with the development of insulin resistance, transplacental transportation of glucose, hyperglucose-mediated coagulation and fibrinolysis disorders in the GDM placenta villi.

## Introduction

Gestational diabetes mellitus (GDM), defined as glucose intolerance that first occurs or is first identified during pregnancy [1], is a common disease that complicates 1–14% of all pregnancies [2]. GDM has negative effects on the mother and the offspring in both short and long term. Women with GDM have a higher risk for developing additional complications during pregnancy, and are more likely to suffer from type-2 diabetes following pregnancy [3], [4]. Offspring of GDM mothers not only have higher morbidity of adverse infant outcomes, but also have an increased risk of diabetes, obesity, and cardiovascular disease in their adult life [5], [6].

Although the etiology of GDM has not been clarified, increasing evidence demonstrates that the placenta, the organ connecting the developing fetus with the maternal body, plays an important role in the development of GDM [7], [8]. In turn, metabolic dysfunctions in GDM, such as hyperglycemia, hyperinsulinemia, and dyslipidemia, can induce structural and functional abnormalities of the placenta villi [9]–[11].

Previous studies have identified a host of gene expression changes in the GDM placenta. These altered genes were generally associated with inflammatory responses, endothelial reorganization [12], cell activation, immune response, organ development, and regulation of cell death [13]. These changes were believed to play a critical role in the interaction between placenta abnormalities and metabolic dysfunction in GDM [8], [12], [13]. However, changes in RNA levels do not conclusively prove interactions since most genes carry out a biological function by producing relative proteins, and RNA level may not fully represent the protein expression level. Therefore, it would be interesting to examine whether the global RNA expression alterations leads to functional proteome changes in the GDM placenta villi. Proteomic approaches have been used previously to analyze protein changes in the placenta from preeclampsia patients [14]. However, to the best of our knowledge, there is no published research regarding the proteome changes in the GDM placenta villi.

Given the clinical significance of GDM, and the power of proteomics approaches, we performed the present study to analyze the proteome changes of GDM placenta villi, to provide evidence for a potential pathway associated with GDM pathogenesis or placental remodeling in GDM.

## Materials and Methods

### 1. Ethics Statement

This study was approved by the research ethical committee of The First Affiliated Hospital of Sun Yat-sen University and written informed consent was provided by all volunteers.

### 2. Subjects

Twenty-five pregnant women complicated with gestational diabetes mellitus were recruited for the GDM group. The diagnosis of GDM was based on American Diabetes Association (ADA) criteria [1]. To prevent potential interference from insulin or metformin, only diet controlled GDM women were recruited for the study. The control group consisted of twenty-five age-matched healthy pregnant women with normal glucose tolerance (NGT) during pregnancy.

### 3. Sample Collection

To improve homogeneity and comparability, placentas selected for the study were from pregnant women that received Cesarean sections. Placenta villi of approximately 3 cm^3^ were obtained from five different intact cotyledons immediately following delivery. After the maternal decidual layer was removed, the tissue was quickly washed with ice-cold PBS, frozen in liquid nitrogen, and stored at −80°C for further protein or mRNA extraction. Samples for immunohistochemistry were fixed in 4% paraformaldehyde for three days and embedded in paraffin before sectioning.

### 4. Protein Preparation for Two-dimensional Electrophoresis

Eight placenta villi samples from the GDM group and eight from the NGT group were randomly chosen for two-dimensional electrophoresis (2DE). For each sample, a total of 300 mg placental tissue was disrupted in liquid nitrogen and solubilized in 1000 µL lysis buffer consisting of 7 M urea, 2 M thiourea, 4% w/v CHAPS, 1% w/v DTT, 2% v/v IPG buffer (pH 3–10), 1% v/v PMSF and 1% v/v protease inhibitor cocktail. After incubating on ice for 2 hours, the sample was centrifuged at 16000 g for 30 min at 4°C to remove debris. The supernatant was collected and further purified using a ReadyPrep 2D Clean-up kit (Bio Rad, Hercules, CA, USA) to improve 2DE gel quality by removing contaminating substances in the sample. Next, the concentrations of the purified protein samples were determined by Bradford protein assay according to the manufacturer’s instructions (Bio-Rad, Hercules, CA, USA), using bovine serum albumin (BSA) as standard. The purified protein samples were stored at −80°C until 2DE was performed.

### 5. Two-dimensional Gel Electrophoresis

The eight purified protein samples from above were pooled to form a final sample for each group. For 2 DE analysis, 1 mg of sample was diluted in sample buffer consisting of 50 mM Tris-HCl (pH 8.5), 7 M urea, 2 M thiourea, 2% CHAPS, 0.3% dithiothreitol (DTT), 0.5% IPG buffer (pH 3–10 linear), and 10 µL of a protease inhibitor mixture to a final volume of 450 µL. Protein samples were applied on immobilized pH 3–10 linear gradient IPG strips (24 cm) (Bio-Rad, Hercules, CA, USA) and active rehydration was performed at 30 V for 6 h then 60 V for 6 h. Focusing was performed using an Ettan IPGphor III IEF system (GE Healthcare, Waukesha, WI, USA) at 200 V for 2 h, 500 V for 2 h, 1000 V for 2 h, 5000 V for 2 h, after which the voltage was gradually increased to and held at 10000V until a total of 90000Vhs was reached, then kept at 500 V for 5 h.

After focusing, strips were equilibrated for 15 min in 75 mM Tris-HCl (pH 8.8), 6 M urea, 2% w/v SDS, 29.3% v/v glycerol, and 1.0% DTT, followed by a 15 min incubation in the same buffer containing 2.5% iodoacetamide in place of DTT. Second-dimension electrophoresis was performed on a 12.5% polyacrylamide gel, using the Ettan DALT Six system (GE Healthcare, Waukesha, WI, USA). Each gel was run at 2w for 1 h at 15°C, and then increased to 15w until the tracking dye migrated to within 1 cm of the bottom of the gel.

### 6. Protein Visualization and Computer Analysis of Protein Spots

2-D gels were fixed in 50% methanol for 2 h, visualized by Coomassie Blue Colloidal Staining (GE Healthcare, Waukesha, WI, USA), and scanned using the ImageScanner system (GE Healthcare, Waukesha, WI, USA) combined with LabScan software(GE Healthcare, Waukesha, WI, USA). All gel images were analyzed using ImageMaster 2D Platinum software (GE Healthcare, Waukesha, WI, USA). Gel images from each group were edited, and spots were matched manually. A unique identification number was assigned to matching spots on different gels. Normalization of the spot intensities was conducted according to the total optical density of the gel. Spots which had more than 2 fold change in relative spot volume were identified as significantly differential spots between gels.

### 7. In-gel Tryptic Digestion

Spots from 2-DE gels selected for further analysis were excised using a blade and gel pieces were transferred to microfuge tubes. After rinsing with distilled water and destaining with potassium ferricyanide and sodium thiosulfate, the gel pieces were dehydrated in 100% acetonitrile. 2 µL (25 ng/µL) of modified porcine trypsin in 25 mM ammonium bicarbonate_,_ pH 8, was added to each sample then incubated at 37°C overnight. The trypsin-digested solutions were collected and the remaining peptides were extracted from the gel pieces by incubating in 0.1% Trifluoroacetic acid/60% acetonitrile for 15 min prior to drying in a vacuum centrifuge.

### 8. MALDI-MS and MS/MS Analysis

MALDI-TOF/TOF mass spectrometry measurements were performed using a Bruker Ultraflex III MALDI-TOF/TOF MS (Bruker Daltonics, Leipzig, Germany) operating in reflectron mode with 20 kV accelerating voltage and 23 kV reflecting voltage. A saturated solution of α-cyano-4-hydroxycinnamic acid in 50% acetonitrile and 0.1% trifluoroacetic acid was used as the matrix. 1 µL of the matrix solution and sample solution at a ratio of 1∶1 was applied onto the Score384 target well. By routine, a standard peptide calibration mix in the mass range 800–3200 Da (Bruker Daltonics, Leipzig, Germany) was analyzed for external calibration of the mass spectrometer. The calibration mix contained: Angiotensin II, Angiotensin I, Substance P, Bombesin, ACTH clip 1–17, ACTH clip 18–39, Somatostatin 28. A series of eight samples were spotted around one external calibration mixture. The SNAP algorithm (S/N threshold: 5; Quality Factor Threshold: 30) in FlexAnalysis 3.0 was used to pick up the 100 most prominent peaks in the mass range m/z 700–4000. The subsequent MS/MS analysis was performed in a data-dependent manner, and the 5 most abundant ions fulfilling certain preset criteria (S/N higher than 3 and Quality Factor higher than 30) were subjected to high energy CID analysis. The collision energy was set to 1 keV, and nitrogen was used as the collision gas.

### 9. Database Searching

Peptide mass fingerprints (PMFs) were searched using the program Mascot 2.1 (Matrix Science Ltd) against the database of SwissProt (version 20091028, 510076 sequences). The search parameters were as follows: trypsin digestion with one missed cleavage; carbamidomethyl modification of cysteine as a fixed modification and oxidation of methionine as a variable modification; peptide tolerance maximum, ±0.3 Da; MS/MS tolerance maximum, ±50 ppm; peptide charge, +1; monoisotopi mass. p<0.05 was used for a local PMF search. For unambiguous identification of proteins, more than 5 peptides must be matched for a PMF search.

### 10. Western Blot Analysis

Western blot on Annexin A2, Annexin A4, Annexin A5, 14-3-3 ζ/δ, and Ras related protein Rap1A was performed on 10 cases (other than the cases for 2DE/MS study) of placenta villi from both the GDM and NGT groups. 30 µg of protein from each sample was loaded onto each lane of 12% resolving and 4% stacking polyacrylamide gels (GE Healthcare, Waukesha, WI, USA) and electrophoresed through a Bio-Rad system (Bio-Rad, Hercules, CA, USA) combined with Laemmli SDS buffering system (25 mM Tris-base, 192 mM glycine, 0.1%SDS). Next, proteins were transferred to PVDF membranes at 70V for 50 min in ice cold transferring buffer and blocked in 5% skimmed milk for 1 h at room temperature. The PVDF membranes were then incubated separately with antibody: rabbit anti-Annexin A2 (Abcam, ab41803, Cambridge, MA, USA, 1 µg/ml), rabbit anti-Annexin A4 (Abcam, ab65846, Cambridge, MA, USA, 0.5 µg/ml), rabbit anti-Annexin A5 (Epitomics, 3225-1, Burlingame, CA, USA, 0.5 µg/ml), rabbit anti-14-3-3 ζ/δ (BioLegend, 614802, San Diego, CA, USA, 1 µg/ml), rabbit anti-Rap (Epitomics, 1726-1, Burlingame, CA, USA, 2 µg/ml), and mouse anti-β-actin (Sigma-Aldrich, A1978, Saint Louis, MO, USA, 0.1 µg/ml ) at 4°C overnight. The membranes were incubated with the indicated secondary antibody for 1 h at room temperature. The immunopositive bands were visualized using Western Lightning chemiluminescence reagents (Invitrogen, Carlsbad, CA, USA). All Western blot exposures were in the linear range of detection, and the intensities of the resulting bands were quantified using Image J software.

### 11. RT and Realtime PCR

Total RNA from placenta villi was extracted using TRIzol reagent (Invitrogen, Carlsbad, CA, USA) according to manufacturer’s instructions, and the concentration and purity of RNA was assessed by NanoDrop® ND-1000 spectrophotometry (Thermo Scientific, Rockford, IL, USA). 2 µg of total RNA was used in the first strand cDNA synthesis with TaqMan® Reverse Transcription Reagents (Takara Bio Inc., Otsu, Shiga, Japan) according to manufacturer’s instructions.

Real-time PCR were performed on 15 samples from each group with the ABI PRISM 7500 Sequence Detection System (Applied Biosystem, Foster City, CA, USA), using SYBR green real-time PCR mix (Takara Bio Inc., Otsu, Shiga, Japan) according to the manufacturer’s specifications. Primers for real-time PCR are shown in Table S1. Reaction conditions were as follows: 95°C for 30 sec, followed by 40 cycles of 95°C for 5 sec and 60°C for 34 sec. Each sample was performed in triplicate in a 25 µL reaction volume, and GAPDH was used as reference gene. Relative quantification of gene expression was performed by the 2^−ΔΔCt^ method based on Ct values for both target and reference genes. Results of real-time PCR analysis are given as mean ± S.E.M.

### 12. Immunohistochemistry

To study the location of Annexin A2, Annexin A4, Annexin A5, 14-3-3 ζ/δ, and Ras related protein Rap1A in human placenta villi, immunohistochemistry (IHC) was performed on samples from each group. The detection of these proteins in the placental tissues was carried out using a two-step IHC procedure. First, paraffin blocks were cut into 5 µm-thick sections. Then, the sections were deparaffinized in xylene and rehydrated in graded alcohol concentrations. Nonspecific binding was blocked by pre-incubation with blocking solution for 5 min, then the sections were incubated for 2 h at room temperature with antibodies against Annexin A2 (10 µg/ml), Annexin A4(5 µg/ml), Annexin A5(5 µg/ml), 14-3-3 ζ/δ (10 µg/ml) and Rap(20 µg/ml). The primary antibodies used for IHC were the same products used in the Western blot analysis. Following incubation with primary antibodies, sections were incubated with appropriate secondary antibody for 1 h. Substrate-chromogen DAB reagent was then added to each section following a rinse, and finally hematoxylin solution was used to stain nuclei.

### 13. Statistics

Data were input and analyzed by SPSS11.0 database. The results were expressed as mean ± SD or mean ± S.E.M, and statistical analysis was carried out using independent student’s t-test. *P* value <0.05 was considered as significant.

## Results

### 1. Baseline Characteristics of the Research Population

Twenty-five pregnant women were recruited for each group. Clinical and laboratory data were compared between pregnant women complicated with gestational diabetes mellitus (GDM) and normal glucose tolerance (NGT). As shown in Table 1, no statistically significant differences can be found between GDM and NGT group in maternal age, parity, gravidity, pre-gravidity and pre-partum BMI, gestational age, newborn gender, and birth length. Pregnant women complicated with GDM have higher glucose levels in each time point of the OGTT test; they also have a higher glycated hemoglobin level and basal insulin concentration than women in the NGT group. GDM mothers have heavier fetuses (at birth) and placentas than their NGT counterparts.

**Table 1:** Baseline clinical characteristics and biochemical parameters of the research population (Mean±SD).

	Gestational diabetes Mellitus (n = 25)	Normal Glucose Tolerance(n = 25)	*P*
*Maternal Parameters*			
Age (year)	31.28±3.13	30.80±3.44	0.608
Gravidity	1.52±0.82	1.60±0.82	0.732
Parity	1.00	1.04±0.20	0.327
Pre-gravidity BMI †(kg/m^2^)	22.13±2.11	21.96±3.55	0.838
Pre-partum BMI (kg/m^2^)	27.83±2.84	27.56±3.46	0.767
OGTT‡-fast (mmol/l)	4.65±0.45	4.21±0.32	<0.001
OGTT-1h (mmol/l)	11.75±2.61	7.97±0.95	<0.001
OGTT-2h (mmol/l)	9.94±1.63	6.33±1.00	<0.001
HbA1c§ (%)	5.94±0.50	4.69±0.44	<0.001
Basal insulin (µU/ml)	16.29±5.89	9.40±2.35	<0.001
*Fetal Parameters*			
Gestational Age (day)	271.64±5.45	273.84±5.37	0.157
Gender			1.000
Male (n, %)	12, 48	12, 48	
Female (n, %)	13, 52	13, 52	
Birth Weight (kg)	3.55±0.33	3.28±0.44	0.018
Birth Length (cm)	50.12±1.45	49.92±1.58	0.643
Placental weight (kg)	0.59±0.043	0.56±0.049	0.026

† BMI: body mass index; ‡OGTT: oral glucose tolerance test; §HbA1c: Glycated hemoglobin.

### 2. Differential Analysis of GDM and NGT Placental Proteomes by 2-DE

**Figure 1:**
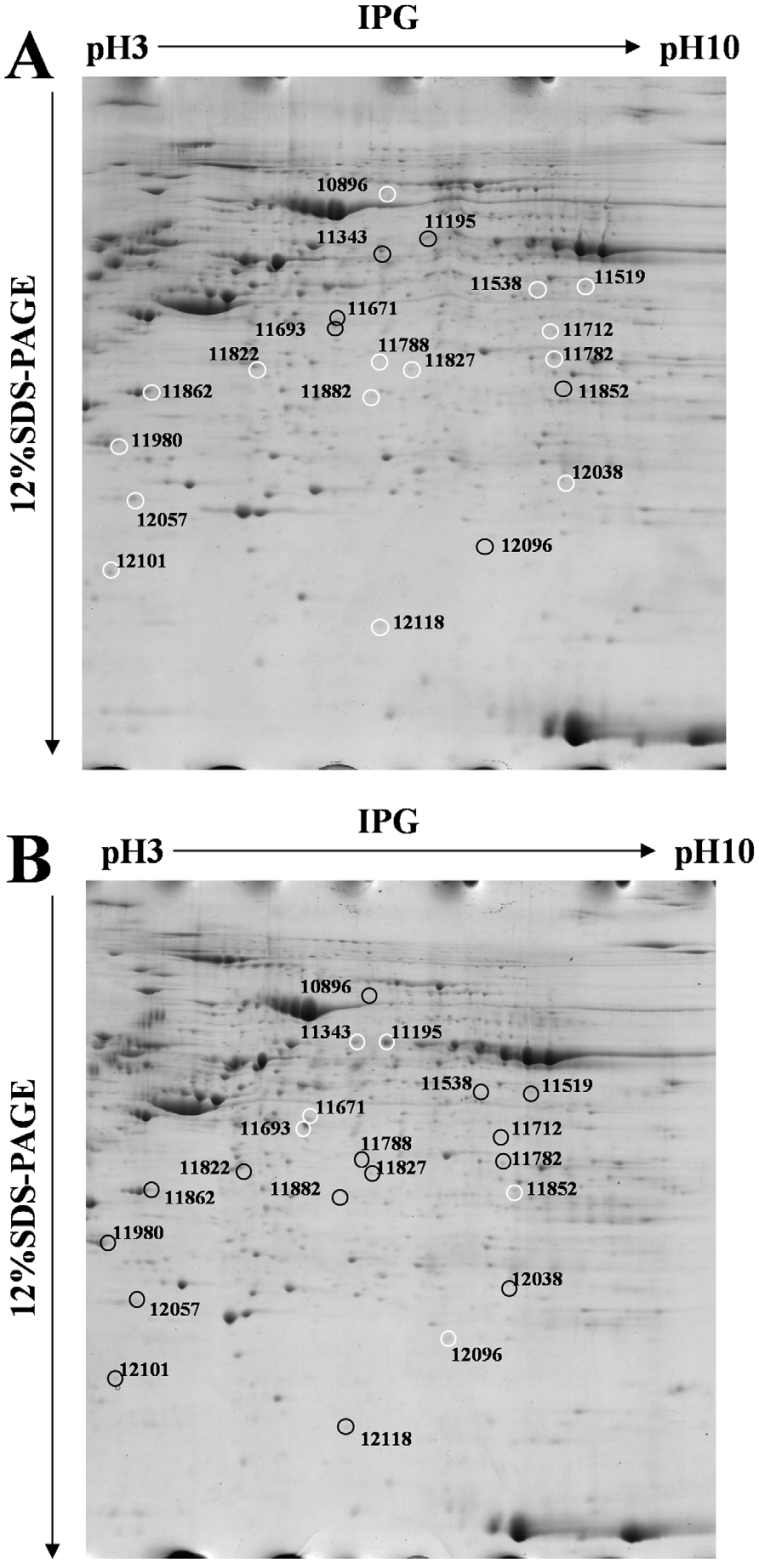
Representative 2DE gel images of placenta villi samples from gestational diabetes mellitus pregnancy and normal glucose tolerance pregnancy. (**A**) Gestational diabetes mellitus pregnancy (GDM) Group. (**B**) Normal glucose tolerance pregnancy (NGT) group. The differentially expressed protein spots are circled on the gels and labeled with unique spot numbers. The spot number on the gels corresponds to the numbers in Table 2. White circles indicate up-regulated proteins in the gels analyzed using ImageMaster Software, while black circles indicate down-regulated proteins.

To investigate the proteomic alterations in the GDM placenta villi, the protein levels in samples from GDM and NGT pregnant women were compared using 2 DE. In order to account for fetal gender differences, placenta villi from four male and four female pregnancies comprised each group. As shown in Fig. 1, approximately 1000 spots were detected on each gel. The ratio of relative spot volume (%Vol) was compared between GDM and NGT groups using ImageMaster software, and 21 protein spots in the 2DE gels were identified as significantly different between the two groups (Fig. 1). Among the 21 protein spots, 15 were up-regulated and 6 were down-regulated in the GDM group. The ratios of relative spot volume (%Vol) for up- and down-regulated spots in the GDM group are summarized in Table 2.

**Table 2:** Identification of differentially expressed proteins in GDM placenta villi.

Spot No§	%Vol Ratio†	Protein ID	Protein name	Mw(kDa)	pI	Sequence coverage (%)	Mascot MS/MS score	Functional association	Subcellular location
*up-regulated proteins in GDM* (GDM/Control)									
11822	1000000	P62879	Guanine nucleotide-binding proteinG(I)/G(S)/G(T) subunit beta-2	37.331	5.60	7	142	Transducer	Cytoplasm
11827	1000000	O00487	26S proteasome non-ATPase regulatory subunit 14	34.577	6.06	7	92	Hydrolase Metalloprotease Protease	Proteasome
11862	1000000	P08758	Annexin A5	35.937	4.94	9	95	Blood CoagulationSignal Transduction	Cytoplasm
11882	1000000	P09525	Annexin A4	35.883	5.85	20	57	Anti-ApoptosisSignal Transduction	Cytoplasm
11980	1000000	P63104	14-3-3 protein zeta/delta	27.745	4.73	9	62	Anti-ApoptosisSignal Transduction	Cytoplasm
10896	2.4378	P41250	Glycyl-tRNA synthetase	83.140	6.61	3	84	ATP Binding	CytoplasmMitochondrion
12101	2.42479	P24844	Myosin regulatory light polypeptide 9	19.827	4.80	12	131	Calcium Ion Binding	Muscle Myosin Complex
11782	2.14439	P07355	Annexin A2	38.604	7.56	13	276	Calcium-Dependent Phospholipid Binding	Basement MembraneExtracellular MatrixSecreted
11519	2.08983	P07954	Fumarate hydratase, mitochondrial	54.637	6.99	1	31	Fumarate Hydratase Activity	Cytoplasm Mitochondrion
*down-regulated proteins in GDM* (Control/GDM)									
11343	2.15177	P23381	Tryptophanyl-tRNA synthetase, cytoplasmic	53.165	5.83	7	146	Tryptophan-Trna Ligase Activity	Cytoplasm
11693	2.20513	P02675	Fibrinogen beta chain	55.928	8.54	6	136	Platelet Activation	External Side Of Plasma Membrane
11671	2.73296	P02675	Fibrinogen beta chain	55.928	8.54	6	150	Platelet Activation	External Side Of Plasma Membrane
11852	2.77444	P02671	Fibrinogen alpha chain	94.973	5.7	1	69	Platelet Activation	External Side Of Plasma Membrane
12096	5.23071	P62834	Ras-related protein Rap-1A	20.987	6.38	15	160	Signal Transduction	Cell Membrane
11195	20.7913	P02675	Fibrinogen beta chain	55.928	8.54	6	108	Platelet Activation	External Side Of Plasma Membrane

§ Spot No is the unique protein spot number referring to the labels in Figure 1.

† %Vol Ratio is analyzed by ImageMaster software and calculated by the ratio of relative spot volume of the two groups.

### 3. Identification of Differentially Expressed Proteins by MALDI-TOF/TOF MS

**Figure 2:**
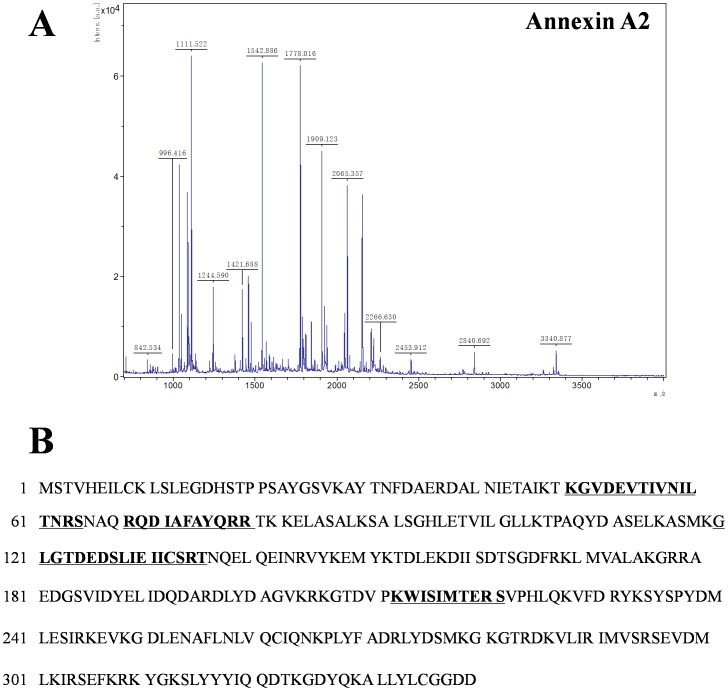
Identification of Annexin A2 by MALDI TOF/TOF mass spectrometry. (**A**) MS/MS spectra of Annexin A2. (**B**) Amino acid sequence of Annexin A2. Underlined bold text indicates matched peptide sequences.

The differentially expressed spots were excised from gels, digested with trypsin and identified by MALDI-TOF/TOF mass spectrometry. The peptide mass fingerprinting (PMF) and MS/MS identification of Annexin A2 by MALDI-TOF-MS/MS is presented in Fig. 2 as a representative example.

As shown in Table 2, 15 of the 21 protein spots were successfully identified: 9 up-regulated and 6 down-regulated (3 different spots were identified as the same protein) in the GDM group. In accordance with UniProt (Swiss-Prot/TrEMBL) and NCBI databases, the up-regulated proteins in the GDM group were mainly involved in anti-coagulation, signal transduction, anti-apoptosis, ATP binding, phospholipid binding, and calcium ion binding, while the down-regulated proteins were associated with platelet activation, tryptophan-tRNA ligase activity, and signal transduction. According to the literature, the identified proteins are mainly expressed in the plasma membrane, cytoplasm, and mitochondrion.

### 4. Verification of Differentially Expressed Proteins by Western Blot Analysis

**Figure 3:**
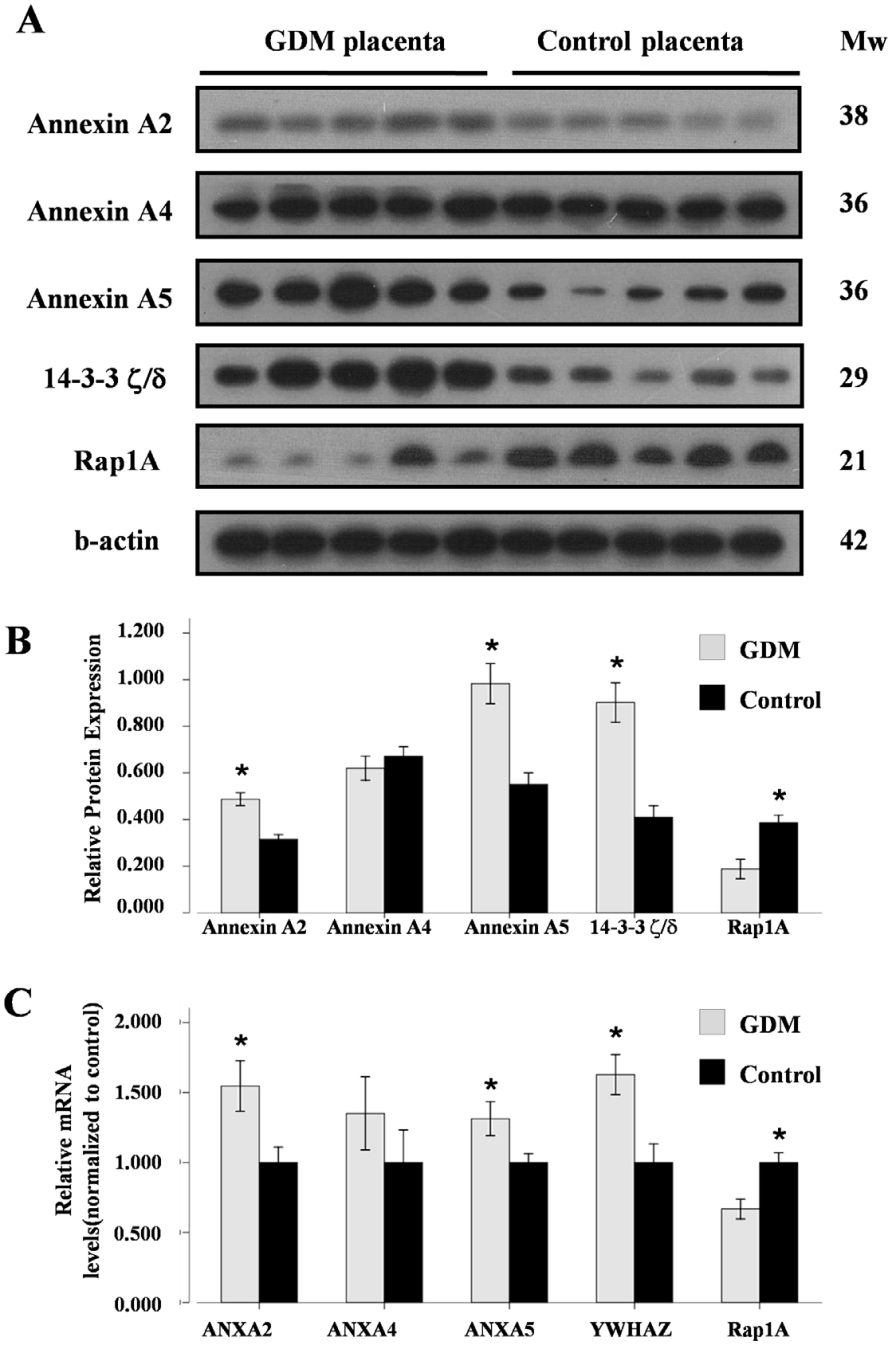
Protein and mRNA expression of Annexin A2, Annexin A4, Annexin A5, 14-3-3 ζ/δ, and Ras related protein Rap1A in placenta villi from GDM and NGT (Control) groups. (**A**) A representative image of Western blots for Annexin A2, Annexin A4, Annexin A5, 14-3-3 ζ/δ, and Ras related protein Rap1A in placenta villi from each group. β-actin was used to verify equivalent loading. (**B**) Graphic representation of relative abundance of Annexin A2, Annexin A4, Annexin A5, 14-3-3 ζ/δ, and Ras related protein Rap1A normalized to β-actin. Data was given as mean ± S.E.M values of ten samples from each group. * P<0.05 by independent student’s t-test between GDM and NGT (Control) group. (**C**) Realtime RT-PCR analysis of relative mRNA levels of the genes from the five identified proteins in placenta villi from GDM and NGT (Control) groups. Bar graph shows mean ± S.E.M of 15 samples from each group with three repeats. ANXA2, ANXA4, ANXA5, YWHAZ, and Ras related protein Rap1A indicate the gene names of Annexin A2, Annexin A4, Annexin A5, 14-3-3 ζ/δ, and Ras related protein Rap1A, respectively.

Western blots were performed to confirm differential protein expression between GDM and NGT groups. Ten placenta villi samples from each group (in addition to the samples for the 2DE/MS study) were obtained for Western blot analysis of five differentially expressed proteins: Annexin A2, Annexin A4, Annexin A5, 14-3-3 ζ/δ, and Ras related protein Rap1A. As shown in Fig. 3 (Panel A and B), GDM placenta villi samples have increased Annexin A2 (1.5 fold change), Annexin A5 (1.8 fold change), and 14-3-3 ζ/δ (2.2 fold change) expression, and decreased Rap1A expression (0.5 fold change), compared with NGT placenta villi. These results were consistent with the 2DE results. However, the protein level of Annexin A4, which showed increased expression in GDM placenta villi by 2DE analysis, was unchanged when analyzed by Western blot.

### 5. Alteration of mRNA Levels of Differentially Expressed Proteins

To compare the mRNA levels of Annexin A2, Annexin A4, Annexin A5, 14-3-3 protein ζ/δ, and Ras related protein Rap1A in GDM and NGT placenta villi, fifteen samples from each group were analyzed by realtime RT-PCR. As shown in Fig. 3 (Panel C), the relative mRNA levels of Annexin A2 (1.5 fold change), Annexin A5 (1.3 fold change), and 14-3-3 ζ/δ (1.6 fold change) were higher, while the relative mRNA level of Rap1A was lower (0.7 fold change) in the GDM group. The mRNA levels of four proteins were significantly different (the increase observed for Annexin A4 was not significant), suggesting that these protein alterations in the GDM placenta villi were partially, if not wholly, due to the changes in mRNA expression.

### 6. Verification of Differentially Expressed Proteins by IHC

**Figure 4:**
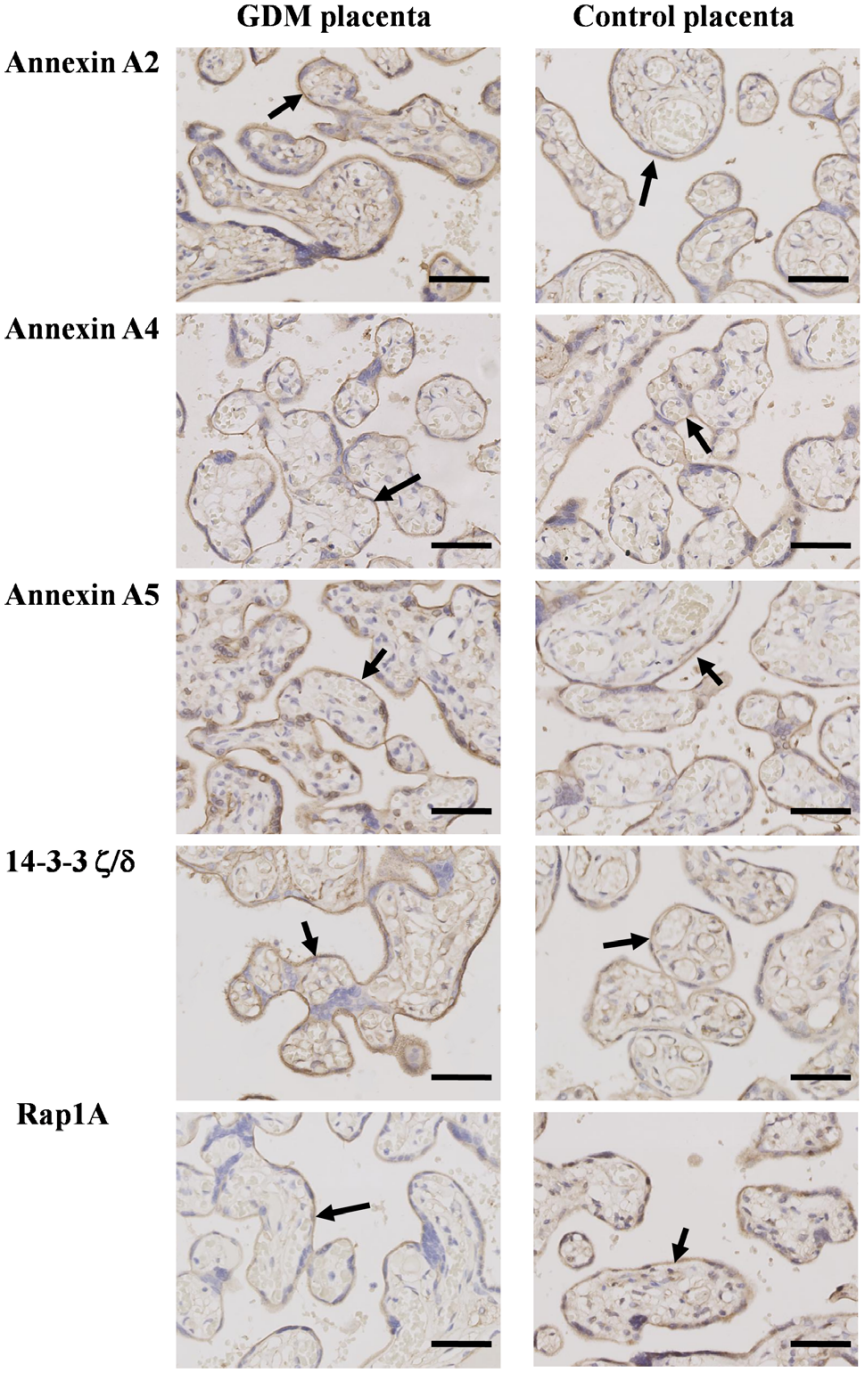
Immunohistochemistry analysis of Annexin A2, Annexin A4, Annexin A5, 14-3-3 ζ/δ, and Ras related protein Rap1A in placenta villi from GDM and NGT (Control) groups. Annexin A2, Annexin A4, Annexin A5, 14-3-3 ζ/δ and Ras related protein Rap1A are mainly expressed in trophoblast cells of placenta villi. Magnification: 200x Arrows: indicate location of each protein. Bar: 50 µm.

To investigate the localization of differentially expressed proteins in the placenta villi, we performed IHC on Annexin A2, Annexin A4, Annexin A5, 14-3-3 ζ/δ, and Ras related protein Rap1A. As shown in Fig. 4, all five proteins were mainly located in trophoblast cells of the placenta villi.

## Discussion

The placenta plays an important role in the development of gestational diabetes mellitus (GDM), while hyperglycemia, hyperinsulinemia, and other metabolic dysfunctions in GDM patients in turn induce structural and functional alterations of the placenta. Previous research has identified changes in placental gene expression in women suffering from GDM [12], [13]. As a polygenic disorder, GDM would therefore likely be associated with a variety of placental protein alterations rather than just a single protein modification. In the present study, we therefore investigated the proteomic differences between GDM and normal placenta villi. Fifteen differentially expressed proteins were successfully identified by mass spectrometry. The molecular functions of these identified proteins include blood coagulation, signal transduction, anti-apoptosis, ATP binding, phospholipid and calcium ion binding, platelet activation, and tryptophan-tRNA ligase activity, consistent with previous research on gene alterations in the GDM placenta villi. The expression levels of five identified proteins were confirmed by Western blot, immunohistochemistry and realtime RT-PCR. Results revealed that Annexin A2, Annexin A5 and 14-3-3 protein were up-regulated, while Ras related protein Rap1A was down-regulated in GDM placenta villi at both the mRNA and protein level.

**Figure 5:**
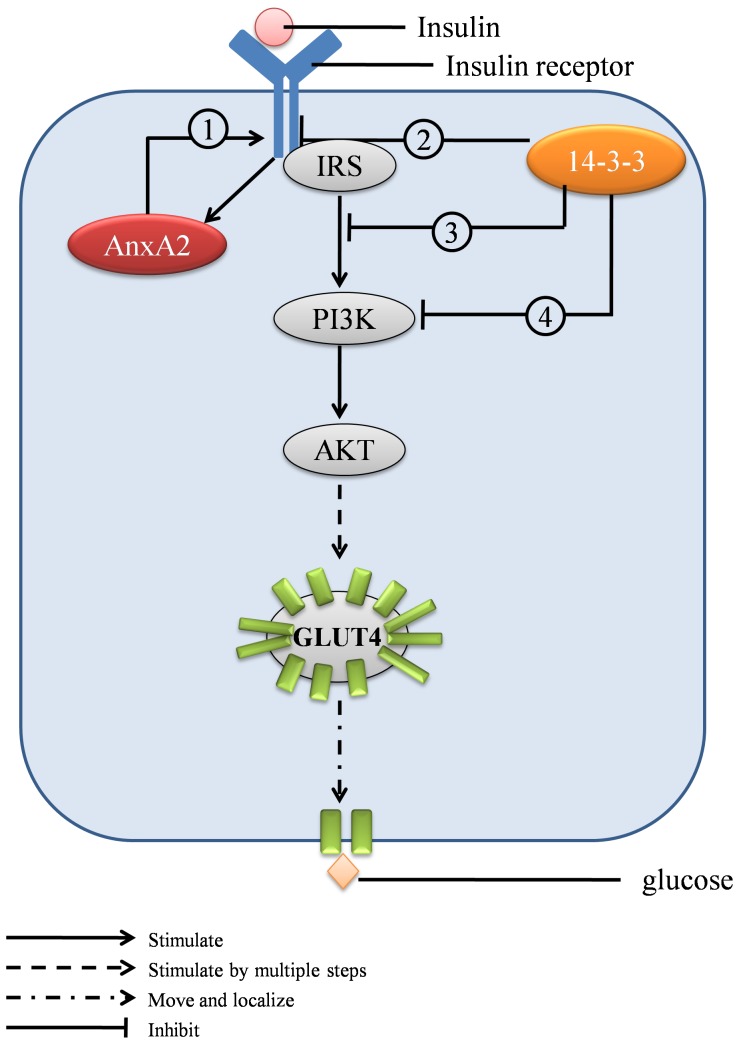
Differentially expressed proteins and insulin resistance. The insulin signal pathway has been previously described by Pessin (Ref. 15). When stimulated by insulin, the insulin receptor activates insulin receptor substrates (IRS), PI3K, AKT and GLUT4 sequentially, leading to the localization of GLUT4 to the cell membrane for transfer of glucose. **1**. Insulin stimulation activates Annexin A2 (Ref. 20) resulting in internalization of the insulin receptor by endosome formation (Ref. 17–20). **2**.14-3-3 proteins compete with IR for IRS binding (Ref. 21). **3**.14-3-3 proteins decrease PI3K activity (Ref. 22). **4**.14-3-3 proteins bind and translocate the IRS/PI3K complex (Ref. 23).

Insulin resistance is the pivotal mechanism of both type 2 diabetes mellitus (T2DM) and GDM. Insulin induces cell glucose uptake by sequentially activating the insulin receptor (IR), insulin receptor substrates (IRS), PI3K, AKT and GLUT4 [15]. Consequently, GLUT4 localizes on the cell membrane and transfers glucose into the cell [15]. Thus, the major causes of insulin resistance include modification or internalization of IR, inhibition of IR/IRS or IRS/PI3K association, and mis-localization of the IRS/PI3K complex or GLUT4, etc [15]. Two proteins involved in the insulin pathway (Annexin A2 and 14-3-3 ζ/δ) were found to be differentially expressed in the present study, and therefore may be directly associated with insulin resistance in GDM (Figure 5).

IR internalization is the major mechanism by which cells maintain the response to insulin stimulation within a normal range [16], and Annexin A2 plays a critical role in IR internalization. Annexin A2 has long been recognized as a major factor in endosome formation [17]–[19], and insulin stimulation is able to activate Annexin A2 [20]. Notably, blocking the activity of Annexin A2 decreases IR internalization [20] and thus, Annexin A2 acts as a very important intermediate in the insulin stimulation/IR internalization feedback loop. Up-regulation of Annexin A2 in GDM placenta villi, as seen in the present study, likely results from overstimulation of the IR due to high levels of extracellular insulin. This in turn increases IR internalization and insulin resistance.

The 14-3-3 proteins are also involved in the insulin signal pathway. Upon stimulation by insulin, the insulin receptor (IR) phosphorylates tyrosine residues in insulin receptor substrate-1 (IRS-1) and subsequently, phosphorylated IRS-1 associates with phosphoinositol-3-kinase (PI3K). 14-3-3 proteins can bind IRS-1 and thus inhibit insulin signaling in several ways. Firstly, 14-3-3 proteins compete with IR for IRS binding [21]. Secondly, the association of 14-3-3 proteins and IRS decreases PI3K activity [22]. Thirdly, binding of the 14-3-3 protein to the IRS-1/PI3K complex, can result in translocation of the complex to the cytosol, thereby impeding the pathway [23]. It is clear from these previous studies that 14-3-3 proteins play an essential role in insulin desensitization and therefore may be associated with insulin resistance induced by a chronic hyperglucose and hyperinsulin milieu. It is therefore likely that up-regulation of this complex in the GDM placenta villi is directly related to hyperinsulinemia in GDM mothers.

Combining the alterations in expression of Annexin A2 and 14-3-3 complex, we speculate that GDM placental cells make adjustments in response to chronic insulin stimulation by down-regulating insulin receptors and blocking the insulin signal pathway, thereby reducing the uptake of glucose.

Hyperglycemia and hyperinsulinemia are two major pathophysiological changes in GDM. Maternal hyperglycemia is able to induce fetal hyperglycemia due to an increased transplacental transfer of glucose, while maternal insulin is not able to cross the placenta. Fetal hyperglycemia can stimulate insulin secretion by the fetal pancreas, subsequently leading to normalization of fetal glycemia. However, intrauterine hyperglycemia and hyperinsulinemia are also associated with macrosomia in GDM pregnancy, by a mechanism known as the Pederson hypothesis [24], [25], the pivotal step of which is the transplacental transportation of high levels of glucose. Although the expression of insulin-sensitive GLUT4 on term placenta is low [26], our finding of the down-regulated insulin signal pathway and putative decreased glucose uptake of placenta villi may still have a potential biological significance in the control of transplacental transportation of glucose, and therefore is important in the counteraction of intrauterine hyperglycemia, hyperinsulinemia and macrosomia in GDM pregnancy.

Proteins found to have altered expression between GDM and NGT placental groups in the present study are associated with additional cellular functions including coagulation and fibrinolysis balance.

**Figure 6:**
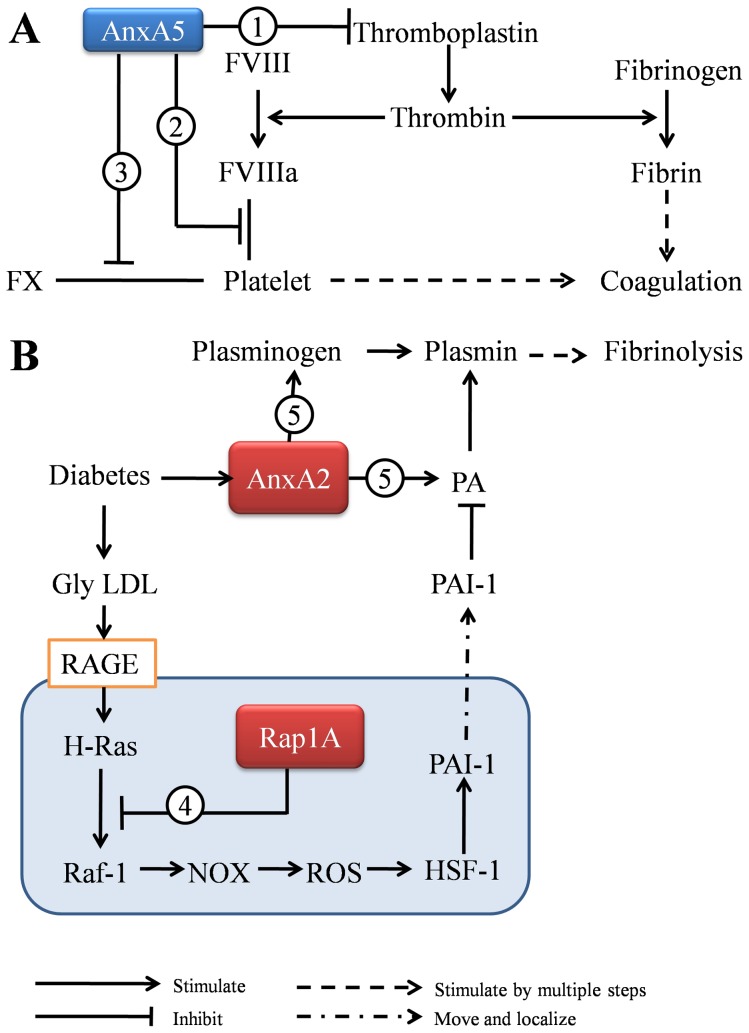
Differentially expressed proteins involved in coagulation and fibrinolysis. (**A**) Annexin A5 inhibits coagulation. The coagulation activation process in this panel has been previously described in Harrison’s Principles of Internal Medicine (ref. 27 ). **1**. Annexin A5 inhibits thromboplastin (Ref. 29). **2**. Annexin A5 competes with factor VIII for phosphatidylserine (PS) binding sites (Ref. 28). **3**. Annexin A5 blocks the binding of factor X to thrombin-stimulated platelets (Ref. 30). (**B**) **Ras related protein Rap1A and Annexin A2 on fibrinolysis.** The fibrinolysis pathway in this panel has been previously described in References 33∼38. **4**. Ras related protein Rap1A inhibits the binding of Ras and Raf in a competitive manner (Ref. 32). Down-regulated Rap1A in GDM placenta villi may decrease the inhibition of the Ras signal pathway, thereby increasing PAI-1 levels. **5**. Annexin A2 increases plasmin levels by binding to tissue plasminogen activator (t-PA) and plasminogen(Ref. 39).

A hypercoagulable state becomes more common during pregnancy, especially in those complicated with GDM. However, we found Annexin A5, a major protein involved in hypocoagulation, was up-regulated in GDM placenta villi. Annexin A5, otherwise known as placenta anticoagulant protein 1, can form a shield around negatively-charged phospholipid molecules and inhibit blood coagulation [27]. The anti-coagulation effects of Annexin A5 includes competing with activated factor VIII for phosphatidylserine (PS) binding sites [28], inhibiting thromboplastin [29] and blocking the binding of factor X to thrombin-stimulated platelets [30] (Figure 6 A). Therefore, the placenta may up-regulate and secrete more Annexin A5 in order to counteract the hypercoagulation state induced by GDM.

Besides hypercoagulation, GDM has long been as associated with an attenuated fibrinolysis state [31]. The present finding of Ras related protein Rap1A down-regulation may be associated with the diminished fibrinolytic activity in these GDM patients (Figure 6 B). As a member of the Ras subfamily, Ras related protein Rap1A can inhibit the binding of Ras and Raf in a competitive manner [32]. Previous studies have reported that glycated low-density lipoprotein (gly-LDL) increases in diabetic patients [33], [34]. Gly-LDL actives RAGE and a cascade including H-Ras, Raf-1, NOX, ROS, and HSF-1, and consequently increases the expression of PAI-1[35]–[37]. Plasminogen activator inhibitor-1(PAI-1), the major inhibitor of the plasminogen activator (PA), is increased and associated with diminished fibrinolytic activity in diabetes [38]. In the Gly-LDL/RAGE pathway, the expression and association of Ras and Raf is a very pivotal step. Thus, our finding of Ras related protein Rap1A down-regulation may contribute to this pathway by decreasing the competitive inhibition of Ras/Raf binding, thereby enhancing the downstream effects. Consequently, down-regulation of Ras related protein Rap1A is associated with low fibrinolytic activity by increasing PAI-1 expression.

On the contrary, Annexin A2 may contribute to enhanced fibrinolysis in the GDM placenta villi. Previous studies suggest Annexin A2 may increase plasmin generation by binding to tissue plasminogen activator (t-PA) and plasminogen [39] and restore impaired fibrinolytic activity with glucose and insulin [40]. As mentioned above, high glucose (diabetes) is typically associated with a low-fibrinolysis state [31], which seems contradictory to our present finding of up-regulated Annexin A2 in the GDM placenta villi. Previous studies showed that Annexin A2 is up-regulated on the surface of endothelial cells by high concentrations of glucose [41] and acts as an intermediate in diabetes-induced enhancement of plasmin activity in the aorta; and thus counteracts the hyper-thrombotic trend in vessels of diabetic patients [40], [41]. Similarly, we hypothesize that the up-regulation of Annexin A2 in the placenta villi is a response to hyperglycemia or a prothrombotic milieu in GDM patients, playing a role to keep the fibrolysis balance in the placenta.

Interestingly, three protein spots (numbers: 11693, 11671, and 11195) were identified as the same protein (fibrinogen beta chain). It is a common phenomenon in 2DE for the same protein to migrate to multiple locations on the gel [42]. Potential reasons include: (1) nonspecific proteolysis or cleavage; (2) post-translational or artifactual modifications or post-translational processing, both of which may alter the molecular weight [42] and isoelectric point [43] of a protein, resulting in multiple protein spots on the gel.

In summary, the present study mainly focuses on placental proteome alterations in pregnant women complicated with GDM, which may either be the cause or result of physiological changes in GDM. Differentially expressed proteins found and identified in the current study include Annexin A2, Annexin A5, 14-3-3 protein ζ/δ, and Ras related protein Rap1A, which are involved in the regulation of the insulin pathway and coagulation/fibrinolysis and may play important roles in insulin resistance and diabetic-related coagulation/fibrinolysis alterations. To the best of our knowledge, the present work is the first proteomic study on GDM placenta villi. Further studies may be performed to uncover additional proteomic changes and to confirm the relationship between alterative proteins and GDM physiology.

## Supporting Information

Table S1 **Primers for real-time PCR.**
(DOC)
