# Supplementary material for: Altered Protein Expression in Gestational Diabetes Mellitus Placentas Provides Insight into Insulin Resistance and Coagulation/Fibrinolysis Pathways
Source: PLoS One. 2012 Sep 7;7(9):e44701. doi: 10.1371/journal.pone.0044701 (PMC3436753; doi:10.1371/journal.pone.0044701)
Supplement: Table S1 — Primers for real-time PCR. (DOC) [file pone.0044701.s001.doc]

Table S1. Primers for real-time PCR

| Primer | Sequences |
| --- | --- |
| *ANXA2* |  |
| Forward primer | ACTTTGATGCTGAGCGGGATG |
| Reverse primer | CGAAGGCAATATCCTGTCTCTGTG |
| *ANXA4* |  |
| Forward primer | GGTAGCTTTGAAGATGCTCTGCTG |
| Reverse primer | GAAGTGTGCCCGGATATCCAAC |
| *ANXA5* |  |
| Forward primer | AAACCATTGACCGCGAGACTTC |
| Reverse primer | GGTCTCTGCAAGGTAGGCAGGTA |
| *YWHAZ* |  |
| Forward primer | CCCAATGCTTCACAAGCAGAGA |
| Reverse primer | GGTATGCTTGTTGTGACTGATCGAC |
| *RAP1A* |  |
| Forward primer | CAGGGCCAGAATTTAGCAAGACA |
| Reverse primer | TGACTATGGGCCTAGAGCAGCA |
| *GAPDH* |  |
| Forward primer | GCACCGTCAAGGCTGAGAAC |
| Reverse primer | TGGTGAAGACGCCAGTGGA |
